# Nighttime intramedullary nailing of femoral fractures is not associated with worse outcomes: a 13-year Level I trauma center cohort

**DOI:** 10.1007/s00402-026-06484-0

**Published:** 2026-08-29

**Authors:** Artsiom Abialevich, Vadim Benkovich, Vladislav Osinsky, Yosef Adiniaev, Ilan Tzaytlin, Yotam Heinemann, Asaf Acker

**Affiliations:** 1 Orthopedic Department, Soroka Medical Center, Beersheba, Israel; 2 Faculty of Health Sciences, Ben-Gurion University of the Negev, Beersheba, Israel; 3 Faculty of Medicine, University of Debrecen, Debrecen, Hungary

**Keywords:** Femoral fractures, Intramedullary nailing, Nighttime surgery, Postoperative complications, Trauma surgery

## Abstract

**Background:**

Whether nighttime orthopedic trauma surgery adversely affects outcomes remains controversial. Evidence specifically addressing intramedullary fixation across femoral fracture locations remains limited. This study evaluated whether nighttime surgery was associated with a higher rate of recorded postoperative complications than daytime surgery.

**Methods:**

This retrospective observational cohort included adult patients with nonperiprosthetic femoral fractures treated with intramedullary fixation between January 2010 and January 2023 at a single Level I trauma center. Of 311 screened records, 23 were excluded because the patient was younger than 18 years (*n* = 17) or the fracture was periprosthetic (*n* = 6), leaving 288 procedures (242 daytime and 46 nighttime). The primary outcome was any recorded postoperative complication during available follow-up. Secondary outcomes included radiographic abnormality, delayed union (> 24 weeks), nonunion, revision surgery, and mortality. Surgeon experience was categorized as senior surgeon or resident. Exploratory fracture-location subgroup analyses and multivariable logistic regression were performed.

**Results:**

Nighttime patients were younger (35.1 ± 19.1 vs. 46.6 ± 24.6 years, *p* < 0.001), more frequently male (82.6% vs. 64.0%, *p* = 0.014), more often injured by a high-energy mechanism (80.4% vs. 56.6%, *p* = 0.002), and underwent surgery sooner after trauma (4.0 [2.0–10.0] vs. 18.0 [7.0–48.0] hours, *p* < 0.001). Residents were the recorded primary surgeon in 54.3% of nighttime and 39.3% of daytime procedures (*p* = 0.057). After adjustment for demographic, injury, fracture-location, time-to-surgery, and surgeon-experience variables, nighttime surgery was not associated with the primary outcome (adjusted odds ratio 0.45, 95% confidence interval 0.15–1.38; *p* = 0.163). Delayed union (28.3% vs. 19.4%, *p* = 0.233) and revision surgery (4.3% vs. 12.4%, *p* = 0.130) did not differ significantly.

**Conclusions:**

In this single-center cohort, nighttime intramedullary fixation of femoral fractures was not associated with higher observed rates of postoperative complications, impaired fracture healing, or revision surgery compared with daytime fixation. These findings suggest that operative timing alone may not be a major determinant of outcome within an organized Level I trauma system. Nevertheless, the small nighttime cohort, unequal group sizes, fracture heterogeneity, and limited statistical precision require cautious interpretation and preclude definitive conclusions regarding equivalence or subgroup-specific safety. Larger multicenter studies with standardized assessment of surgeon experience, staffing, fatigue, and fracture-specific outcomes are warranted.

## Introduction

Femoral fractures occur after high-energy trauma in younger adults and fragility-related mechanisms in older patients and constitute an important component of orthopedic trauma workload [1]. Intramedullary fixation is a standard treatment for many proximal, shaft, and distal femoral fracture patterns and aims to restore alignment and stability while facilitating mobilization [2]. In patients with multiple injuries, operative timing is also influenced by physiologic status and resuscitation priorities [3].

As trauma services provide continuous coverage, femoral fracture fixation is often performed outside routine daytime hours because of clinical urgency, operating room availability, and institutional workflow [4]. Concerns regarding nighttime surgery include reduced staffing, limited senior availability, and fatigue-related impairment of surgical and anesthesiology performance [5].

Previous research has produced inconsistent findings. Some authors report comparable outcomes after daytime and after-hours orthopedic trauma surgery [6, 7], whereas others describe higher complication rates or mortality after out-of-hours procedures [8]. Hip fracture evidence is similarly mixed: systematic and nationwide analyses have not consistently demonstrated worse outcomes [9, 10], while other studies have identified procedure- or setting-specific differences [11, 12].

Dedicated orthopedic trauma operating rooms may reduce delays and after-hours workload, although their influence on complications depends on the surrounding trauma system and staffing model [13, 14]. Evidence specifically evaluating intramedullary fixation across the spectrum of adult femoral fractures remains limited.

The objective of this study was to compare recorded perioperative, healing, and revision outcomes after daytime and nighttime intramedullary fixation of femoral fractures and to assess whether operative timing remained associated with postoperative complications after accounting for surgeon experience and measured case-mix differences.

## Materials and methods

**Fig. 1:**
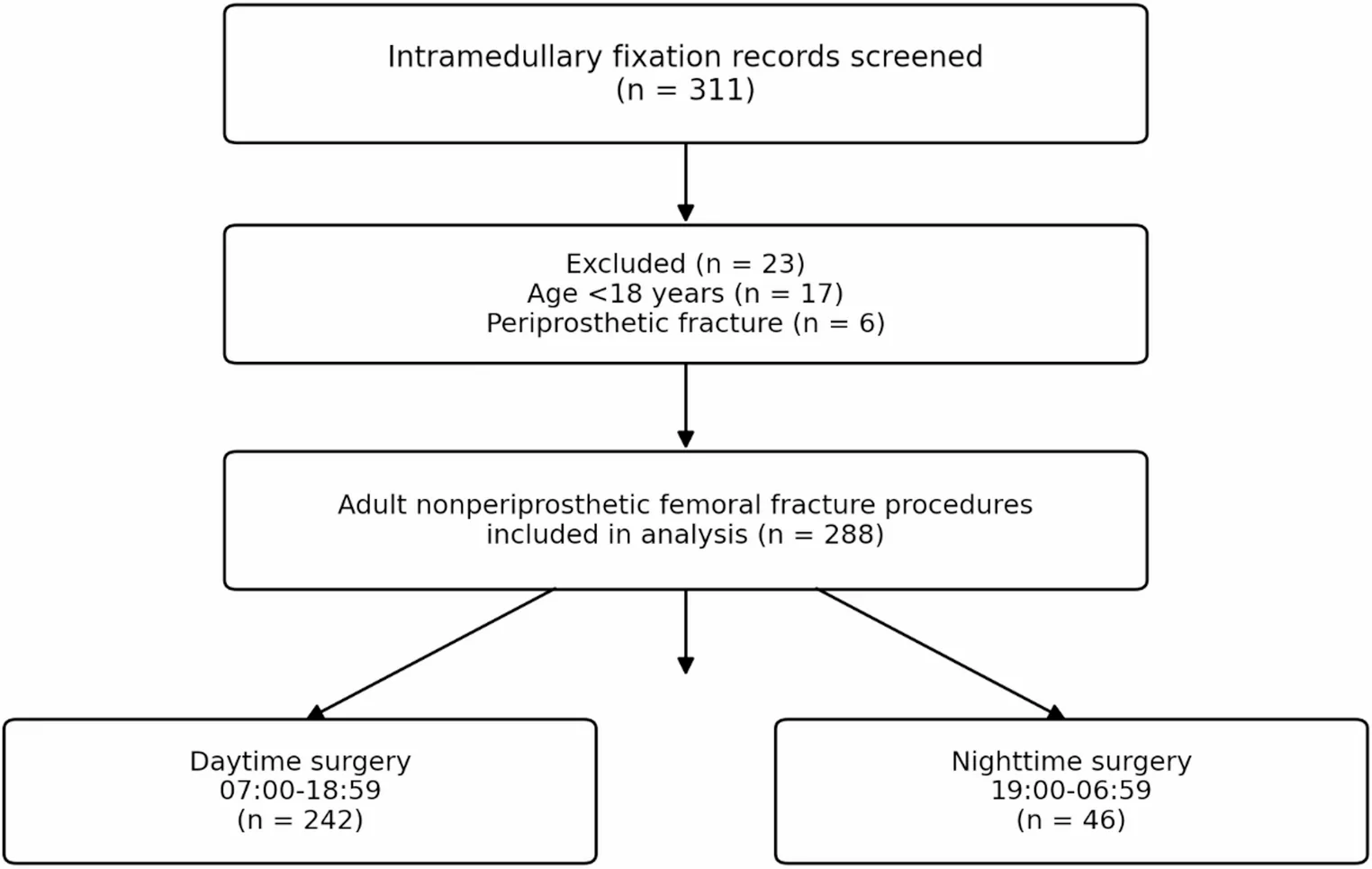
STROBE-style patient selection flowchart

This retrospective observational cohort study was conducted at a single Level I trauma center after institutional review board approval (SOR-0089-26). Institutional trauma and operative databases were screened for femoral fracture procedures treated with intramedullary fixation between January 2010 and January 2023. The screened dataset contained 311 records. Records were excluded after identification when the patient was younger than 18 years (*n* = 17) or the fracture was periprosthetic (*n* = 6). The final cohort therefore comprised 288 adult nonperiprosthetic femoral fracture procedures (Fig. 1). The manuscript does not classify procedures managed exclusively with plates or external fixation as exclusions because such procedures were outside the intramedullary-fixation sampling frame.

Procedures were stratified by recorded surgical start time, defined by skin incision, into daytime (07:00–18:59) and nighttime (19:00–06:59) groups. Demographic variables included age, sex, body mass index (BMI), documented diabetes mellitus, smoking or tobacco use, alcohol use, corticosteroid exposure, prior surgery on the same limb, fragility-fracture history, and previous infection or osteomyelitis. Injury variables included fracture location, AO/OTA classification, energy of injury, open or closed status, initial displacement, and time from trauma to surgery.

Fracture location was assigned using the recorded fracture location, fracture type, and AO/OTA code. AO/OTA 31 fractures were classified as intertrochanteric; fractures explicitly recorded as subtrochanteric without a 31 code were classified as subtrochanteric; AO/OTA 33 or explicitly distal fractures were classified as distal femur; remaining fractures were classified as femoral shaft. Surgeon experience was recorded as senior surgeon or resident. Operative duration, anesthesia type, regional block, and recorded preoperative and intraoperative complications were also extracted.

The primary outcome was any recorded postoperative complication during the available follow-up period. Secondary outcomes included a combined documented radiographic abnormality variable because the source dataset recorded radiolucency or malalignment in a single field, infection, deep vein thrombosis (DVT), pulmonary embolism (PE), nonunion, delayed union, revision surgery, and mortality during recorded follow-up. Delayed union was defined as radiographic union later than 24 weeks. Radiographic union time and follow-up duration were analyzed as continuous variables.

Continuous variables were summarized as mean±standard deviation or median (interquartile range), as appropriate. Between-group comparisons used Welch’s t test for approximately normally distributed continuous variables and the Mann-Whitney U test for skewed variables. Categorical variables were compared using the chi-square test or Fisher’s exact test when cell counts were small. Exploratory analyses were repeated within intertrochanteric, subtrochanteric, femoral shaft, and distal femur strata; these analyses were considered descriptive because of the small nighttime sample in several strata.

A multivariable logistic regression model evaluated the association between nighttime surgery and the primary outcome using complete cases (*n* = 286). Covariates were age, sex, open fracture, high-energy mechanism, surgeon experience, time from trauma to surgery, and fracture location. Results are presented as adjusted odds ratios (aORs) with 95% confidence intervals (CIs). Statistical significance was defined as *p* < 0.05. No adjustment for multiple comparisons was applied to exploratory subgroup analyses.

## Results

Of 311 screened intramedullary-fixation records, 17 patients younger than 18 years and 6 patients with periprosthetic fractures were excluded. The final cohort included 288 procedures: 242 daytime and 46 nighttime procedures (Fig. 1).

Nighttime patients were younger than daytime patients (35.1 ± 19.1 vs. 46.6 ± 24.6 years, *p* < 0.001) and were more frequently male (82.6% vs. 64.0%, *p* = 0.014). BMI did not differ. Documented diabetes was present in 34 daytime patients (14.0%) and 3 nighttime patients (6.5%; *p* = 0.229). Smoking or tobacco use, alcohol use, corticosteroid exposure, prior same-limb surgery, fragility-fracture history, and prior infection or osteomyelitis were not significantly different (Table 1).

Femoral shaft fractures predominated in both groups. Nighttime procedures were more frequently associated with high-energy trauma (80.4% vs. 56.6%, *p* = 0.002) and occurred sooner after injury (median 4.0 [2.0–10.0] vs. 18.0 [7.0–48.0] hours, *p* < 0.001). Open-fracture prevalence did not differ significantly. Three fractures recorded as AO/OTA 32 C were treated at night and none during daytime (*p* = 0.004); other individual AO/OTA categories did not differ significantly (Table 2).

Operative duration and anesthesia distribution were similar. Residents were the recorded primary surgeon in 25/46 nighttime procedures (54.3%) and 95/242 daytime procedures (39.3%), a difference that did not reach statistical significance (*p* = 0.057). Recorded preoperative and intraoperative bleeding and neurovascular complications were uncommon and did not differ between groups (Table 3).

**Fig. 2:**
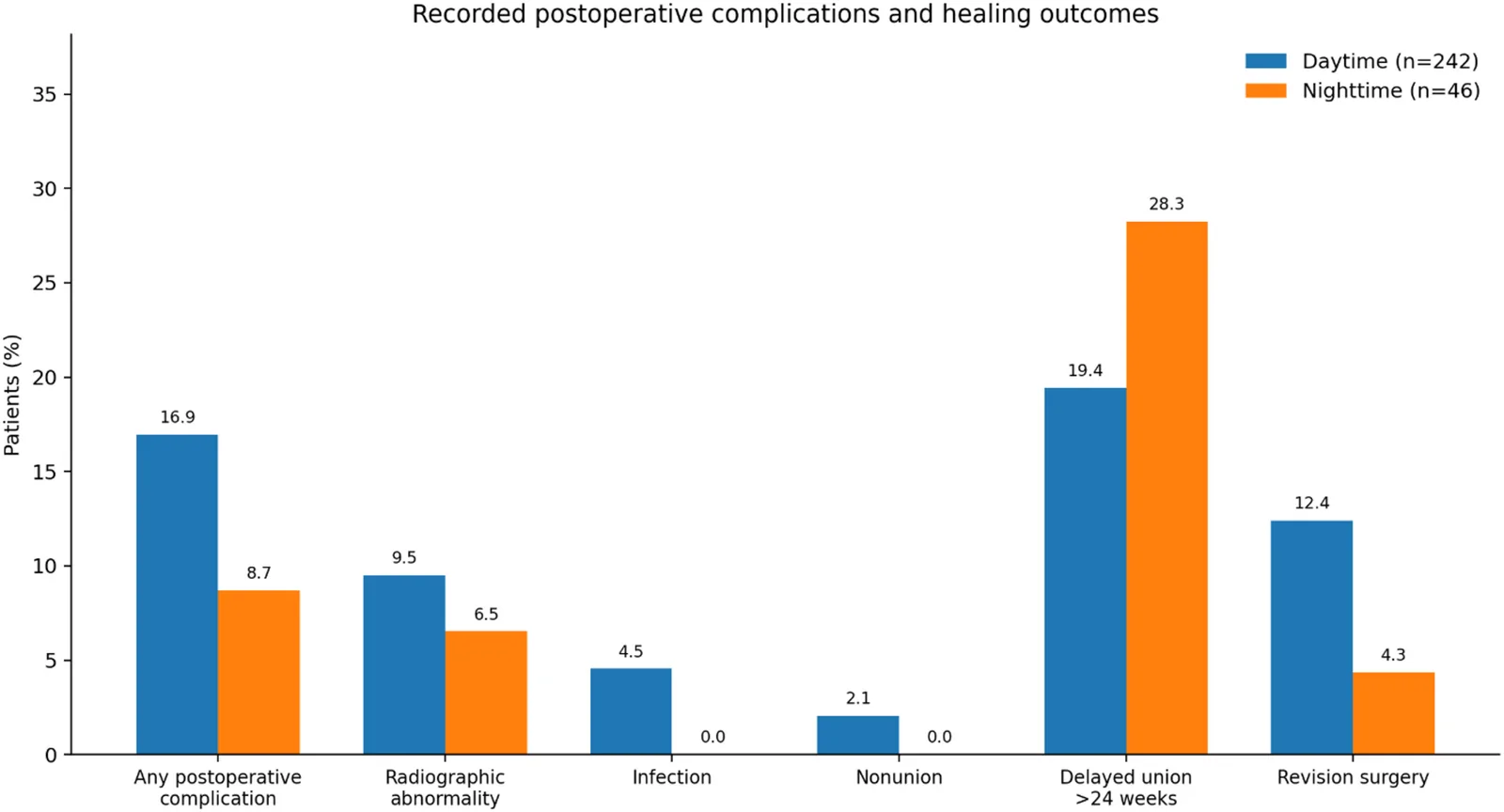
Comparative plot of recorded postoperative complications and healing outcomes

Any recorded postoperative complication occurred in 41 daytime procedures (16.9%) and 4 nighttime procedures (8.7%; *p* = 0.189). Documented radiographic abnormality, infection, nonunion, and delayed union did not differ significantly. Delayed union occurred in 47 daytime procedures (19.4%) and 13 nighttime procedures (28.3%; *p* = 0.233). Revision surgery was recorded after 30 daytime procedures (12.4%) and 2 nighttime procedures (4.3%; *p* = 0.130). Median follow-up was 10.0 months in both groups (*p* = 0.397) (Table 4; Fig. 2). Mortality during recorded follow-up was lower in the nighttime group on unadjusted analysis (2.2% vs. 12.4%, *p* = 0.038), but the nighttime group was substantially younger and this endpoint was not time-standardized.

Exploratory fracture-location analyses were limited by very small nighttime samples (intertrochanteric *n* = 1, subtrochanteric *n* = 2, and distal femur *n* = 4). Within the femoral shaft stratum, nighttime patients were younger (*p* = 0.002), had less documented diabetes (*p* = 0.036), and more often sustained high-energy trauma (*p* = 0.006). No statistically significant differences in the primary outcome or revision surgery were identified within any location stratum, but the subgroup confidence and precision were insufficient for equivalence claims (Table 5).

In the multivariable complete-case model, nighttime surgery was not associated with any recorded postoperative complication (aOR 0.45, 95% CI 0.15–1.38; *p* = 0.163). Resident surgeon status was also not associated with the primary outcome (aOR 1.05, 95% CI 0.53–2.07; *p* = 0.889). The wide CI for nighttime surgery remained compatible with both a clinically meaningful reduction and a clinically meaningful increase in risk.

**Table 1:** Patient demographics and baseline characteristics

Variable	Daytime (*n* = 242)	Nighttime (*n* = 46)	*p* value
*General demographics*			
Age, years	46.6 ± 24.6	35.1 ± 19.1	< 0.001
Sex, male/female	155/87	38/8	0.014
Body mass index, kg/m²	31.3 ± 8.0	32.9 ± 7.8	0.218
*Health factors*			
Documented diabetes mellitus	34 (14.0%)	3 (6.5%)	0.229
Smoking/tobacco use	62 (25.6%)	13 (28.3%)	0.716
Alcohol use	0 (0.0%)	1 (2.2%)	0.160
Corticosteroid exposure	3 (1.2%)	0 (0.0%)	1.000
Previous surgery on the same limb	37 (15.3%)	2 (4.3%)	0.058
History of fragility fracture	4 (1.7%)	0 (0.0%)	1.000
History of infection or osteomyelitis	2 (0.8%)	0 (0.0%)	1.000
Bone-density–affecting medication	1 (0.4%)	0 (0.0%)	1.000

**Table 2:** Fracture and injury characteristics

Variable	Daytime (*n* = 242)	Nighttime (*n* = 46)	*p* value
*Fracture location*			
Intertrochanteric	8 (3.3%)	1 (2.2%)	1.000
Subtrochanteric	4 (1.7%)	2 (4.3%)	0.246
Femoral shaft	193 (79.8%)	39 (84.8%)	0.429
Distal femur	37 (15.3%)	4 (8.7%)	0.241
*AO/OTA classification*			
32 A	67 (27.7%)	17 (37.0%)	0.218
32A1 spiral	13 (5.4%)	2 (4.3%)	1.000
32A2 oblique	13 (5.4%)	1 (2.2%)	0.706
32A3 transverse	28 (11.6%)	5 (10.9%)	1.000
32B	2 (0.8%)	0 (0.0%)	1.000
32B2 intact wedge	42 (17.4%)	7 (15.2%)	0.833
32B3 fragmentary wedge	14 (5.8%)	3 (6.5%)	0.741
32 C	0 (0.0%)	3 (6.5%)	0.004
32C2 intact segmental	5 (2.1%)	0 (0.0%)	1.000
32C3 fragmentary segmental	30 (12.4%)	5 (10.9%)	1.000
*Injury characteristics*			
Low-energy mechanism	105 (43.4%)	9 (19.6%)	0.002
High-energy mechanism	137 (56.6%)	37 (80.4%)	0.002
Closed fracture	186 (76.9%)	30 (65.2%)	0.095
Open fracture	56 (23.1%)	16 (34.8%)	0.095
Initial displacement	203 (83.9%)	39 (84.8%)	0.879
Time from trauma to surgery, hours	18.0 (7.0–48.0)	4.0 (2.0–10.0)	< 0.001

**Table 3:** Surgical and perioperative data

Variable	Daytime (*n* = 242)	Nighttime (*n* = 46)	*p* value
Operative time, minutes	133.0 (94.0-190.2)	151.5 (110.0-183.2)	0.544
*Anesthesia*			
General anesthesia	215 (88.8%)	43 (93.5%)	0.345
Spinal anesthesia	28 (11.6%)	3 (6.5%)	0.311
Regional block	17 (7.0%)	2 (4.3%)	0.748
*Surgeon experience*			
Senior surgeon	147 (60.7%)	21 (45.7%)	0.057
Resident surgeon	95 (39.3%)	25 (54.3%)	0.057
*Preoperative complications*			
Bleeding/blood loss	25 (10.3%)	2 (4.3%)	0.275
Neurovascular injury	5 (2.1%)	2 (4.3%)	0.310
*Intraoperative complications*			
Bleeding/blood loss	30 (12.4%)	4 (8.7%)	0.621
Neurovascular injury	0 (0.0%)	0 (0.0%)	1.000

**Table 4:** Postoperative course and outcomes

Variable	Daytime (*n* = 242)	Nighttime (*n* = 46)	*p* value
Immediate weight bearing	105 (43.4%)	19 (41.3%)	0.794
Delayed weight bearing	137 (56.6%)	27 (58.7%)	0.794
Documented radiographic abnormality	23 (9.5%)	3 (6.5%)	0.779
Any recorded postoperative complication	41 (16.9%)	4 (8.7%)	0.189
Infection	11 (4.5%)	0 (0.0%)	0.222
DVT	0 (0.0%)	0 (0.0%)	1.000
PE	0 (0.0%)	0 (0.0%)	1.000
Nonunion	5 (2.1%)	0 (0.0%)	1.000
Delayed union (> 24 weeks)	47 (19.4%)	13 (28.3%)	0.233
Radiographic union time, weeks	15.0 (11.0–19.0)	14.0 (11.0–26.0)	0.953
Follow-up duration, months	10.0 (5.0–16.0)	10.0 (4.0–12.0)	0.397
Revision surgery	30 (12.4%)	2 (4.3%)	0.130
Mortality during recorded follow-up	30 (12.4%)	1 (2.2%)	0.038

**Table 5:** Exploratory stratified comparison by fracture location

Variable	Intertrochanteric (*n* = 8/1)	Subtrochanteric (*n* = 4/2)	Femoral shaft (*n* = 193/39)	Distal femur (*n* = 37/4)
Age, years	D: 69.9 ± 21.9 N: 74.0 p = NE	D: 29.8 ± 9.5 N: 26.5 ± 6.4 *p* = 0.652	D: 45.3 ± 24.5 N: 34.2 ± 18.6 *p* = 0.002	D: 50.4 ± 23.9 N: 38.5 ± 22.3 *p* = 0.376
Male sex	D: 5/8 N: 0/1 *p* = 0.444	D: 3/4 N: 2/2 *p* = 1.000	D: 126/193 N: 32/39 *p* = 0.058	D: 21/37 N: 4/4 *p* = 0.143
BMI, kg/m²	D: 31.5 ± 8.3 N: 25.2 p = NE	D: 32.1 ± 11.6 N: 34.6 ± 0.7 *p* = 0.696	D: 31.0 ± 8.0 N: 32.6 ± 8.0 *p* = 0.266	D: 32.7 ± 7.8 N: 36.7 ± 7.5 *p* = 0.378
Diabetes	D: 2/8 N: 1/1 *p* = 0.333	D: 0/4 N: 0/2 *p* = 1.000	D: 28/193 N: 1/39 *p* = 0.036	D: 4/37 N: 1/4 *p* = 0.418
Smoking/tobacco	D: 2/8 N: 0/1 *p* = 1.000	D: 2/4 N: 1/2 *p* = 1.000	D: 51/193 N: 11/39 *p* = 0.844	D: 7/37 N: 1/4 *p* = 1.000
High-energy mechanism	D: 2/8 N: 0/1 *p* = 1.000	D: 4/4 N: 1/2 *p* = 0.333	D: 113/193 N: 32/39 *p* = 0.006	D: 18/37 N: 4/4 *p* = 0.111
Open fracture	D: 0/8 N: 0/1 *p* = 1.000	D: 3/4 N: 2/2 *p* = 1.000	D: 40/193 N: 11/39 *p* = 0.297	D: 13/37 N: 3/4 *p* = 0.281
Resident surgeon	D: 4/8 N: 1/1 *p* = 1.000	D: 1/4 N: 1/2 *p* = 1.000	D: 78/193 N: 20/39 *p* = 0.219	D: 12/37 N: 3/4 *p* = 0.130
Any postoperative complication	D: 2/8 N: 0/1 *p* = 1.000	D: 1/4 N: 0/2 *p* = 1.000	D: 30/193 N: 3/39 *p* = 0.313	D: 8/37 N: 1/4 *p* = 1.000
Revision surgery	D: 1/8 N: 0/1 *p* = 1.000	D: 1/4 N: 0/2 *p* = 1.000	D: 20/193 N: 1/39 *p* = 0.216	D: 8/37 N: 1/4 *p* = 1.000

Values are mean±standard deviation for continuous variables and events/total for categorical variables. Subgroup p values are exploratory and were not adjusted for multiple comparisons

*D* daytime, *N* nighttime

## Discussion

The principal finding of this study is that nighttime intramedullary fixation was not associated with a statistically detectable increase in the composite of recorded postoperative complications after adjustment for measured case mix and surgeon experience. This finding should not be interpreted as proof of equivalence. Only 46 procedures occurred at night, the adjusted 95% CI was wide (0.15–1.38), and the fracture-location subgroups contained very few nighttime cases.

Operative timing in trauma is inseparable from injury urgency and patient physiology. Early total care and damage-control concepts emphasize physiologic readiness rather than clock time alone [15]. Contemporary registry analyses similarly show that fixation strategy and timing are influenced by overall injury burden and associated chest trauma [16]. In the present cohort, nighttime patients were younger, more likely to have high-energy injuries, and taken to surgery sooner, demonstrating substantial selection and workflow differences between groups.

Fatigue remains a biologically plausible concern. Experimental studies have shown impaired cognitive and psychomotor performance after sleep deprivation [17, 18]. However, clinical outcomes also depend on supervision, team organization, case selection, and institutional safeguards. We added surgeon experience to the analysis because training level may influence operative duration or reoperation in some femoral-fracture settings [19, 20]. Residents performed a greater proportion of nighttime procedures in our cohort, although the difference was not statistically significant, and resident status was not associated with the primary outcome after adjustment. These findings do not address the potential effect of individual fatigue, as hours awake, preceding workload, and sleep duration were not measured. In addition, this was a resident-performed cohort, with all procedures undertaken without direct intraoperative supervision by senior surgeons.

Prior hip-fracture studies have often reported no major difference between daytime and after-hours surgery, although results vary by design, outcome, and healthcare system [10, 11, 21, 22]. The present study extends this question to a broader femoral-fracture population, but that broader scope also introduces heterogeneity. The intertrochanteric, subtrochanteric, shaft, and distal groups differ in patient age, injury mechanism, biology of union, and operative complexity. The exploratory subgroup analysis therefore serves primarily to expose sampling imbalance rather than to establish subgroup-specific safety.

Temporal variation has also been examined beyond time of day. Trauma studies have assessed possible changes in preventable complications at the start of the academic year [23], and large surgical datasets have identified seasonal variation in postoperative outcomes [24]. In addition, registry evidence shows that the incidence and composition of severe trauma vary according to time, date, and season [25]. These observations suggest that apparent clock-time effects may partly reflect changes in case mix, volume, staffing, and institutional workflow.

Evidence from other healthcare settings likewise suggests that nighttime risk is context dependent. Shift work has been associated with a higher risk and severity of occupational injury among hospital employees [26]. Worse outcomes have also been reported for in-hospital cardiac arrest during nights and weekends [27] and for some patients admitted to intensive care outside routine daytime hours [28]. Although these findings cannot be directly extrapolated to femoral fracture fixation, they emphasize that temporal risk may arise from the broader care environment rather than from operative timing alone.

Conversely, prospective European data on nonelective surgery found that nighttime operation was not independently associated with in-hospital mortality after risk adjustment [29]. Similarly, nighttime surgery was not associated with a higher rate of intraoperative adverse events in a separate analysis [30]. These findings are consistent with the possibility that organized emergency surgical systems can attenuate time-of-day effects.

However, temporal associations are not uniform across procedures. After-hours colorectal surgery has been associated with increased anastomotic leakage [31], and late operating-room start times for nonemergent cardiac surgery have been linked to higher mortality and cost [32]. Such differences underscore that the consequences of nighttime surgery may depend on procedure complexity, urgency, staffing, and postoperative resource requirements.

Operator experience has also been examined as a potential influence on the clinical course after proximal femoral fracture surgery [33]. This reinforces the importance of accounting for experience, although the present dataset could not quantify individual case volume, technical proficiency, fatigue, or the experience of the remaining operating-room team.

System organization may mitigate temporal risk. Dedicated orthopedic trauma operating rooms can improve access and efficiency [13, 14], while standardized perioperative pathways may reduce variability between daytime and nighttime care. However, these organizational characteristics were not directly quantified in the present study. Furthermore, all procedures were performed by residents without direct intraoperative supervision by senior surgeons, and information regarding the experience, workload, or fatigue of anesthesiologists, nurses, surgical assistants, and scrub staff was unavailable.

The study did not use a uniform 90-day healing endpoint; median recorded follow-up was approximately 10 months, and delayed union was defined as union later than 24 weeks. This is more appropriate for femoral shaft healing than a 90-day endpoint. Even so, follow-up was variable, event timing was not uniformly coded, and mortality was not time-standardized.

Clinical implications should remain limited. The current data do not support delaying an otherwise indicated procedure solely because it begins after 19:00, but they also do not demonstrate that daytime and nighttime surgery are equivalent. Decisions should continue to incorporate patient physiology, fracture urgency, available expertise, and the capability of the entire trauma team.

This study has several limitations. It was retrospective, single-center, and based on a small and imbalanced nighttime cohort. The fracture population was heterogeneous, and the intertrochanteric, subtrochanteric, and distal nighttime subgroups were too small for reliable inferential comparison. Although surgeon experience was recorded, surgeon fatigue, hours worked, sleep duration, supervision, and case volume were not measured. The experience and fatigue of anesthesiologists, nurses, surgical assistants, and scrub staff were also unavailable. Complication timing was not uniformly available, follow-up duration varied, and mortality was not standardized to a fixed interval. Residual confounding by injury severity, physiologic status, associated injuries, and institutional workflow remains possible. Absence of statistically significant between-group differences should be interpreted as a lack of evidence of increased risk rather than as confirmation that nighttime and daytime surgery are equivalent.

## Conclusion

In this single-center cohort, nighttime intramedullary fixation of femoral fractures was not associated with higher observed rates of postoperative complications, impaired fracture healing, or revision surgery compared with daytime fixation. These findings suggest that operative timing alone may not be a major determinant of outcome within an organized Level I trauma system. Nevertheless, the small nighttime cohort, unequal group sizes, fracture heterogeneity, and limited statistical precision require cautious interpretation and preclude definitive conclusions regarding equivalence or subgroup-specific safety. Larger multicenter studies with standardized assessment of surgeon experience, staffing, fatigue, and fracture-specific outcomes are warranted.

## Data Availability

No datasets were generated or analysed during the current study.

## References

[1] Court-Brown CM, Bugler KE, Clement ND, Duckworth AD, McQueen MM (2012) The epidemiology of open fractures in adults. A 15-year review. Injury 43(6):891–897. 10.1016/j.injury.2011.12.007 22204774

[2] Neumann MV, Südkamp NP, Strohm PC (2015) Management of femoral shaft fractures. Acta Chir Orthop Traumatol Cech 82(1):22–32 25748658

[3] Vallier HA, Wang X, Moore TA, Wilber JH, Como JJ (2013) Timing of orthopaedic surgery in multiple trauma patients: development of a protocol for early appropriate care. J Orthop Trauma 27(10):543–551. 10.1097/BOT.0b013e31829efda1 23760182

[4] Baldock TE, Walshaw T, Walker R et al (2023) The ORthopaedic Trauma Hospital Outcomes—patient operative delays (ORTHOPOD) study. Bone Jt Open 4(6):463–471. 10.1302/2633-1462.46.BJO-2023-0040.R1 37350770 PMC10289117

[5] Sturm L, Dawson D, Vaughan R et al (2011) Effects of fatigue on surgeon performance and surgical outcomes: a systematic review. ANZ J Surg 81(7–8):502–509. 10.1111/j.1445-2197.2010.05642.x 22295360

[6] Ricci WM, Gallagher B, Brandt A, Schwappach J, Tucker M, Leighton R (2009) Is after-hours orthopaedic surgery associated with adverse outcomes? A prospective comparative study. J Bone Joint Surg Am 91(9):2067–2072. 10.2106/JBJS.H.00661 19723981

[7] Patel NM, Yoon RS, Koerner JD, Donegan DJ, Liporace FA (2014) Timing of diaphyseal femur fracture nailing: is the difference night and day? Injury 45(3):546–549. 10.1016/j.injury.2013.10.018 24238854

[8] Halvachizadeh S, Teuber H, Cinelli P, Allemann F, Pape HC, Neuhaus V (2019) Does the time of day in orthopedic trauma surgery affect mortality and complication rates? Patient Saf Surg 13:8. 10.1186/s13037-019-0186-4 30766615 PMC6362600

[9] Kim RG, An VVG, Petchell JF (2021) Hip fracture surgery performed out-of-hours: a systematic review and meta-analysis. Injury 52(4):664–670. 10.1016/j.injury.2021.02.049 33648740

[10] Forssten MP, Mohammad Ismail A, Borg T et al (2022) The consequences of out-of-hours hip fracture surgery: insights from a retrospective nationwide study. Eur J Trauma Emerg Surg 48(2):709–719. 10.1007/s00068-021-01804-y 34622327 PMC9001198

[11] Karagoz B, Keceli O, Cukurlu M, Agir I (2022) Comparison of daytime and after-hours surgical treatment of femoral neck fractures. Niger J Clin Pract 25(11):1846–1852. 10.4103/njcp.njcp_285_22 36412292

[12] Chacko AT, Ramirez MA, Ramappa AJ, Richardson LC, Appleton PT, Rodriguez EK (2011) Does late night hip surgery affect outcome? J Trauma 71(2):447–453. 10.1097/TA.0b013e3182231ad7 21825947

[13] Brusalis CM, Shah AS, Luan X, Lutts MK, Sankar WN (2017) A dedicated orthopaedic trauma operating room improves efficiency at a pediatric center. J Bone Joint Surg Am 99(1):42–47. 10.2106/JBJS.16.00640 28060232

[14] Sheasley JA, Faino A, Gupta A, Bompadre V, Schmale GA (2024) Advantages of a dedicated orthopaedic trauma room for children with fractures of the femur treated at a pediatric community hospital. J Pediatr Soc North Am 10:100137. 10.1016/j.jposna.2024.100137 40433580 PMC12088346

[15] Pape HC, Tornetta P 3rd, Tarkin I, Tzioupis C, Sabeson V, Olson SA (2009) Timing of fracture fixation in multitrauma patients: the role of early total care and damage control surgery. J Am Acad Orthop Surg 17(9):541–549. 10.5435/00124635-200909000-00001 19726738

[16] Keß A, Kleber C, Lefering R et al (2025) Reassessing the role of external fixation in polytrauma and severely injured patients with chest trauma and femur fracture: a propensity score-matched analysis from the TraumaRegister DGU. Eur J Trauma Emerg Surg 51:296. 10.1007/s00068-025-02956-x 40986042 PMC12457497

[17] Gerdes J, Kahol K, Smith M, Leyba MJ, Ferrara JJ (2008) The effect of fatigue on cognitive and psychomotor skills of trauma residents and attending surgeons. Am J Surg 196(6):813–820. 10.1016/j.amjsurg.2008.07.030 19095094

[18] Kahol K, Leyba MJ, Deka M et al (2008) Effect of fatigue on psychomotor and cognitive skills. Am J Surg 195(2):195–204. 10.1016/j.amjsurg.2007.10.004 18194679

[19] Toci GR, Stambough JB, Martin JR, Mears SC, Saxena A, PPFF Consortium, Lichstein PM (2023) Effect of fracture type, treatment, and surgeon training on reoperation after Vancouver B periprosthetic femur fractures. J Arthroplasty 38(9):1864–1868. 10.1016/j.arth.2023.03.024 36933681

[20] Forsbacka N, Kolari T, Talme M, Bister V (2023) Surgical residents’ results seem to be non-inferior compared with more experienced surgeons in femoral neck fracture osteosynthesis. Indian J Orthop 57(12):2018–2023. 10.1007/s43465-023-00992-6 38026839 PMC10673761

[21] Rashid RH, Zubairi AJ, Slote MU, Noordin S (2013) Hip fracture surgery: does time of the day matter? A case-controlled study. Int J Surg 11(9):923–925. 10.1016/j.ijsu.2013.07.003 23872033

[22] Switzer JA, Bennett RE, Wright DM et al (2013) Surgical time of day does not affect outcome following hip fracture fixation. Geriatr Orthop Surg Rehabil 4(4):109–116. 10.1177/2151458513518344 24600531 PMC3943363

[23] Inaba K, Recinos G, Teixeira PG et al (2010) Complications and death at the start of the new academic year: is there a July phenomenon? J Trauma 68(1):19–22. 10.1097/TA.0b013e3181b88dfe 20065752

[24] Englesbe MJ, Pelletier SJ, Magee JC et al (2007) Seasonal variation in surgical outcomes as measured by the American College of Surgeons-National Surgical Quality Improvement Program. Ann Surg 246(3):456–465. 10.1097/SLA.0b013e31814855f2 17717449 PMC1959349

[25] Pape-Köhler CI, Simanski C, Nienaber U, Lefering R (2014) External factors and the incidence of severe trauma: time, date, season and moon. Injury 45(Suppl 3):S93–S99. 10.1016/j.injury.2014.08.027 25284243

[26] Horwitz IB, McCall BP (2004) The impact of shift work on the risk and severity of injuries for hospital employees. Occup Med (Lond) 54(8):556–563. 10.1093/occmed/kqh093 15385648

[27] Peberdy MA, Ornato JP, Larkin GL et al (2008) Survival from in-hospital cardiac arrest during nights and weekends. JAMA 299(7):785–792. 10.1001/jama.299.7.785 18285590

[28] Cavallazzi R, Marik PE, Hirani A, Pachinburavan M, Vasu TS, Leiby BE (2010) Association between time of admission to the ICU and mortality: a systematic review and meta-analysis. Chest 138(1):68–75. 10.1378/chest.09-3018 20418364

[29] van Zaane B, van Klei WA, Buhre WF et al (2015) Nonelective surgery at night and in-hospital mortality: prospective observational data from the European Surgical Outcomes Study. Eur J Anaesthesiol 32(7):477–485. 10.1097/EJA.0000000000000256 26001104

[30] Eskesen TG, Peponis T, Saillant N et al (2018) Operating at night does not increase the risk of intraoperative adverse events. Am J Surg 216(1):19–24. 10.1016/j.amjsurg.2017.10.026 29106826

[31] Komen N, Dijk JW, Lalmahomed Z et al (2009) After-hours colorectal surgery: a risk factor for anastomotic leakage. Int J Colorectal Dis 24(7):789–795. 10.1007/s00384-009-0692-4 19301016 PMC2689358

[32] Yount KW, Lau CL, Yarboro LT et al (2015) Late operating room start times impact mortality and cost for nonemergent cardiac surgery. Ann Thorac Surg 100(5):1653–1659. 10.1016/j.athoracsur.2015.04.131 26209491 PMC4630095

[33] Canal C, Kaserer A, Ciritsis B, Simmen HP, Neuhaus V, Pape HC (2018) Is there an influence of surgeon’s experience on the clinical course in patients with a proximal femoral fracture? J Surg Educ 75(6):1566–1574. 10.1016/j.jsurg.2018.04.010 29699929

